# Defluorination of Aqueous Perfluorooctanesulfonate by Activated Persulfate Oxidation

**DOI:** 10.1371/journal.pone.0074877

**Published:** 2013-10-07

**Authors:** Shewei Yang, Jianhua Cheng, Jian Sun, Yongyou Hu, Xiaoyan Liang

**Affiliations:** 1 Ministry of Education Key Laboratory of Pollution Control and Ecological Remediation for Industrial Agglomeration Area, College of Environment and Energy, South China University of Technology, Guangzhou, Guangdong, China; 2 State Key Lab of Pulp and Paper Engineering, College of Light Industry and Food Science, South China University of Technology, Guangzhou, Guangdong, China; Queen's University Belfast, United Kingdom

## Abstract

Activated persulfate oxidation technologies based on sulfate radicals were first evaluated for defluorination of aqueous perfluorooctanesulfonate (PFOS). The influences of catalytic method, time, pH and K_2_S_2_O_8_ amounts on PFOS defluorination were investigated. The intermediate products during PFOS defluorination were detected by using LC/MS/MS. The results showed that the S_2_O_8_^2−^ had weak effect on the defluorination of PFOS, while the PFOS was oxidatively defluorinated by sulfate radicals in water. The defluorination efficiency of PFOS under various treatment was followed the order: HT (hydrothermal)/K_2_S_2_O_8_ > UV (ultraviolet)/K_2_S_2_O_8_ > Fe^2+^/K_2_S_2_O_8_ > US (ultrasound)/K_2_S_2_O_8_. Low pH was favorable for the PFOS defluorination with sulfate radicals. Increase in the amount of S_2_O_8_^2−^ had positive effect on PFOS defluorination. However, further increase in amounts of S_2_O_8_^2−^ caused insignificant improvement in PFOS defluorination due to elimination of sulfate radicals under high concentration of S_2_O_8_^2−^. CF_3_(CF_2_)_n_COOH (n = 0–6) were detected as intermediates during PFOS defluorination. Sulfate radicals oxidation and hydrolysis were the main mechanisms involved in defluorination process of PFOS.

## Introduction

Perfluorooctane sulfonate (C_8_F_17_SO_3_^−^, PFOS) have been widely used in industry as emulsifying agents, surface treatment agents, food containers, fabrics, paper coatings, waxes, fire-fighting foams and polishes [1]. Because of specific characteristics such as unique high surface activity, thermal and acid resistance, and hydro- and lipophobic properties [2], PFOS are considered almost un-degradable in nature. Most conventional degradation technologies are ineffective for degradation of aqueous PFOS since they are inherently recalcitrant to chemical and microbiological treatment [3]–[6]. Even Advanced oxidation processes (AOPs), which utilize the hydroxyl radical, such as UV/O_3_, O_3_/H_2_O_2_, UV/H_2_O_2_ or Fenton's reagent, are also relatively ineffective in PFOS destruction [7]. In fact, the decomposition resistance of PFCs to conventional AOTs is evidenced by the use of PFOS as a surfactant to increase the adsorption of organic pollutants on TiO_2_ to obtain the accelerated AOT effects [2]. Direct photolysis [8], VB_12_/Ti^3+^reduction [9] and permanganate oxidation [2] also show low defluorination efficiencies and incompletely mineralization of PFOS. In contrast, photo reduction [10], [11], sonolysis [12]–[17], sub-critical elemental iron reduction [18], electrochemical oxidation [19] and alkaline ozonation [20] have been proved effective.

Persulfate anion (S_2_O_8_^2−^) is a strong oxidizing agent with a redox potential of 2.0 V, and can be reduced to sulfate anions as shown below (Eq.1) [21]:
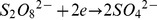
(1)


S_2_O_8_^2−^ can be activated to produce sulfate radicals (SO_4_^•−^) with higher redox potential of 2.6–3.1 V, which are very reactive with a wide range of contaminants. There are two general ways of activating S_2_O_8_^2−^: homolysis of the peroxide bond using heat, ultrasound or light (Eq.2) and an oxidation reduction process (analogous to the Fenton reaction) with electron donors, including e^−^ (aq) from radiolysis of water or low-valent metals such as Fe^2+^ and Ag^+^ (Eq.3).
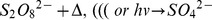
(2)

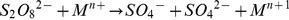
(3)


Previous studies show that SO_4_^•−^ is more prone to redox reactions than hydroxyl radicals (•OH) under neutral conditions, as summarized in the following reactions (Eq.4–6):
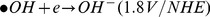
(4)

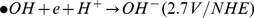
(5)

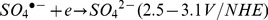
(6)


SO_4_^•−^ is a strong oxidizing radicals and has been known to react with electron-rich moieties through several reaction pathways, including electron exchange, hydrogen abstraction, and direct oxygen transfer. Because of comparative stability of persulfate, strong oxidation characteristics, and pH-independent effectiveness, persulfate and sulfate radical oxidation have been utilized for the oxidative degradation of a number of organics [22]–[26]. It also had been reported that perfluorooctanoic acid (C_7_F_15_COOH, PFOA) and other short-chain perfluorocarboxylic acid (C_n_F_2n+1_COOH, PFCAs) could be decomposed by sulfate radicals [27]–[30]. The results show that PFOA and other PFCAs were effectively decomposed to F^−^ and CO_2_ by using SO_4_^•−^, and almost all of the initial S_2_O_8_^2−^ was transformed to SO_4_^2−^ during the reaction. However, no studies have been reported on persulfate oxidation of PFOS and perfluoroalkyl sulfonate (C_n_F_2n+1_SO_3_^−^).

The present study made first attempt to evaluate the PFOS defluorination effect of S_2_O_8_^2−^ or SO_4_^•−^ using the activated K_2_S_2_O_8_ oxidation systems under air atmosphere. The influence of catalytic method (ultraviolet, ultrasound, hydrothermal, ferrous ion), time, pH and K_2_S_2_O_8_ concentration on PFOS defluorination were investigated. The concentrations of intermediate products were detected using LC/MS/MS. Finally, the possible defluorinaiton mechanism of PFOS with sulfate radicals was proposed.

## Materials and Methods

### Materials

PerfluorooctaneSulfonate (PFOS, C_8_F_17_SO_3_K, 98%), Perfluorooctanoic acid (PFOA, C_7_F_15_COOH, 97%), Perfluoroheptanoic acid (PFHpA, C_6_F_13_COOH, 98%), Tridecafluoroheptanoic acid (PFHxA, C_5_F_11_COOH, 98%), perfluoropentanoic acid (PFPA, C_4_F_9_COOH, 97%) and perfluorobutyric acid (PFBA, C_3_F_7_COOH, 98%) and pentafluoropropionic acid (PFPrA, C_2_F_5_COOH, 97%) was purchased from Sigma (USA). Trifluoroacetic acid (TFA, CF_3_COOH, 99%) was purchased from Sigma (USA). Perfluorohexanesulfonate (PFHxS, C_6_F_13_SO_3_K, 98%), Nonafluorobutanesulfonate (PFBS, C_4_F_9_SO_3_K, 98%) were purchased form TCI(Japan). And ammonium acetate (>99%) and LC-MS grade methanol (HR-GC, >99.99%) were obtained from Merck Chemicals (Germany). Sodium carbonate (Na_2_CO_3_), Sodium bicarbonate (NaHCO_3_), Potassium persulfate (K_2_S_2_O_8_, 98%), Ferrous chloride (FeCl_2_, 98%), Hydrogen chloride (HCl) and sodium hydroxide (NaOH) were purchased from Guangzhou Chemical Reagent Factory(Guangzhou, China). Milli-Q water prepared by Millipore with a conductivity of 18.2 mΩ-cm at 25°C was used in all experiments. All aqueous solutions were prepared with ultrapure water prepared using a Thermo Barnstead Nanopure Diamond water purification system.

### Methods

**Figure 1:**
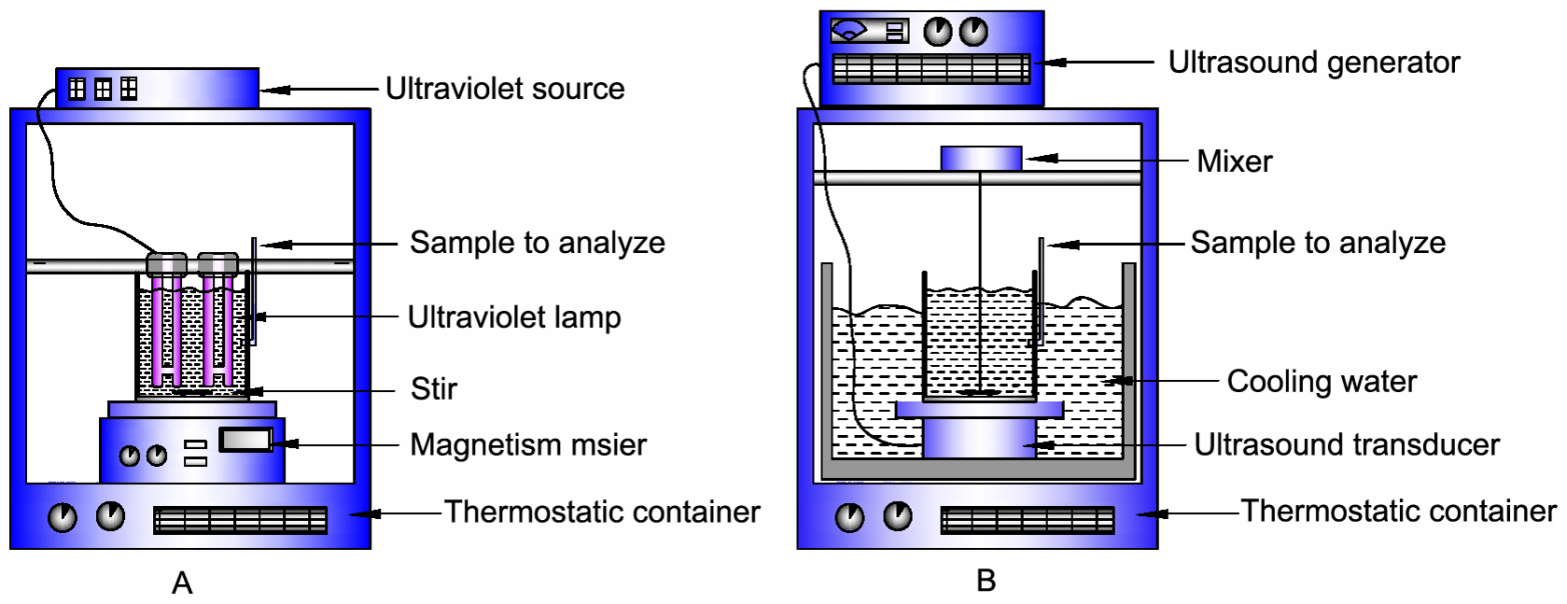
Schematic representation of reactor. (A) UV/K_2_S_2_O_8_. (B) US/K_2_S_2_O_8_.

PFOS stock solution (100 mg/L) was prepared with ultrapure water and then stored in a refrigerator (5°C) before used. The reactions were conducted in batch experiments in polytetrafluoroetylene (PTFE) reactor, due to their superior non-sorption property. Ultraviolet (UV)-activated K_2_S_2_O_8_ oxidation system was shown in Fig. 1A: Two 15W low-pressure mercury lamps (Direct-immersion, H model, SUNSHINE, China) emitting light at 254 nm were placed in the reactor. The photoelectric conversion efficiency was 35%∼38%. The effective radiating length of the UV tube was 9.5 cm (with a radiating surface area of 113.04 cm^2^) and UV fluence rate was around 40 mW/cm^2^. During the irradiation, the UV device was switched on for the first 15 min to warm up the UV lamps. The reactor was placed on constant temperature magnetic stirrer at desired temperature during the reaction time. Ultrasound (US)-activated K_2_S_2_O_8_ oxidation system was shown in Fig. 1B: The ultrasonic apparatus consisted of an ultrasonic generator and an oscillator operated at an applied (calorimetric) power of 100 (86)W (TOSO model, China, 40 kHz). The average power density delivered to the reactor was 2W/cm^2^. The solution temperature was maintained by a thermostatic container. A sink was placed in the a thermostatic container to keep the temperature of both ultrasound transducer and reactor. During the US process, the thermostatic container device was switched on for the first about 20 to 30 min to keep desired temperature (20°C). According to results of control experiments, the temperature of reaction solution remained about 20°C, was no more than 21°C during the reaction (measured by using mercury's thermometer). Hydrothermal (HT)-activated K_2_S_2_O_8_ oxidation system: The reactor was placed on constant temperature magnetic stirrer at desired temperature during the reaction time. And the reactor was closed during the reaction process to prevent loss of H_2_O and gas product by volatilization. Ferrous ion (Fe^2+^)-activated K_2_S_2_O_8_ oxidation system: the addition quantity of Fe^2+^ catalyst was 3 mM. The reactor was placed on constant temperature magnetic stirrer at desired temperature during the reaction time.

In all K_2_S_2_O_8_ oxidation systems, an aqueous (Milli-Q) solution (100 mL) of PFOS (100 mg/L; 0.186 mM) were introduced into the reactor. The samples were withdrawn from the reactor at fixed time intervals and quickly quenched in iced water to end reaction [28], [29]. Control experiments also had been conducted to make sure the reactions could be quenched in iced water (Fig. S1). The PFOS and F^−^ concentrations were determined by LC/MS/MS and IC, respectively. All the results discussed in this article were average values form at least two experiments. The initial pH values were adjusted at about 7.0 by using standard NaOH and HCl solutions except for “solution pH effect”.

LC/MS/MS. The concentrations of PFOS and the intermediates were measured using liquid chromatography tandem mass spectrometry. Liquid chromatography was performed on an HPLC apparatus equipped with an Agilent model 1200 series (RRLC/6410B Triple Quard MS, USA) and was used for the LC separation of PFOS. The HPLC separation was carried out at 30°C using a gradient composed of solution A (10 mM ammonium acetate solution adjusted to pH 4 with the addition of acetic acid) and solvent B (acetonitrile, GR). The gradient expressed as changes in solvent B was as follows: 0 to 2.0 min, a linear increase from 10% to 20% B; 2.0 to 4.0 min, 20% to 45% B; 4.0 to 5.0 min, 45% to 60% B; 5.0 to 6.0 min, 60% to 95% B; 6.0 to 6.1 min, 95% B to 10% B, hold at 4 min. The flow rate was 0.3 mL/min. Ionization was achieved by electro spray in the negative-ion mode. The electro spray conditions were as follows: nitrogen curtain gas flow: 10.0 L/min; gas temperature: 350°C, nebulizer pressure: 275.8 kPa (40.0psi), capillary voltage: 1000 V. The LC/MS/MS acquisition was performed in the multiple reaction monitor (MRM) mode by following the reactions m/z 499.0–79.9, which are characteristic of PFOS. The sonochemical products of PFOS were also measured by LC/MS/MS. The LC and electrospray conditions were the same as those used in the PFOS analysis. The LC/MS/MS acquisition was performed in MRM mode by following the reaction m/z 413.0–368.8 (PFOA), m/z 363.0–318.9 (PFHpA), m/z 313.0–268.8 (PFHxA), m/z 263.0–218.9 (PFPA), m/z 213.0–168.9 (PFBA), m/z 163.0–118.9 (PFPrA), m/z 113.0–68.9 (TFA), m/z 398.9–98.9 (PFHxS) and m/z 298.9–79.9 (PFBS). Mass spectrometry acquisition parameters of the intermediates were shown in Table 1.

**Table 1:** Mass spectrometry acquisition parameters of the intermediates. * Quantitative product ion.

No.	Compound	t_R_(min)	Prec Ion(m/z)	Prod Ion (m/z)	Frag(V)	Cllision energy(V)	Dwell (ms)
1	PFOA	6.45	413.0	368.8*, 168.8	58	0, 12	80
2	PFHxS	6.61	398.9	98.8*, 79.9	129	40, 52	80
3	PFHpA	6.07	363.0	318.9*, 168.8	56	0, 12	80
4	PFHxA	5.59	313.0	268.8*,168.8	62	0, 12	80
5	PFBS	5.66	298.9	98.8, 79.9*	114	28, 36	80
6	PFPA	4.85	263.0	218.9*	50	0	80
7	PFBA	2.92	213.0	168.9*	55	4	80
8	PFPrA	1.47	163.0	118.9*	60	4	80
9	TFA	1.39	113.0	68.9*	60	4	80

**Figure 2:**
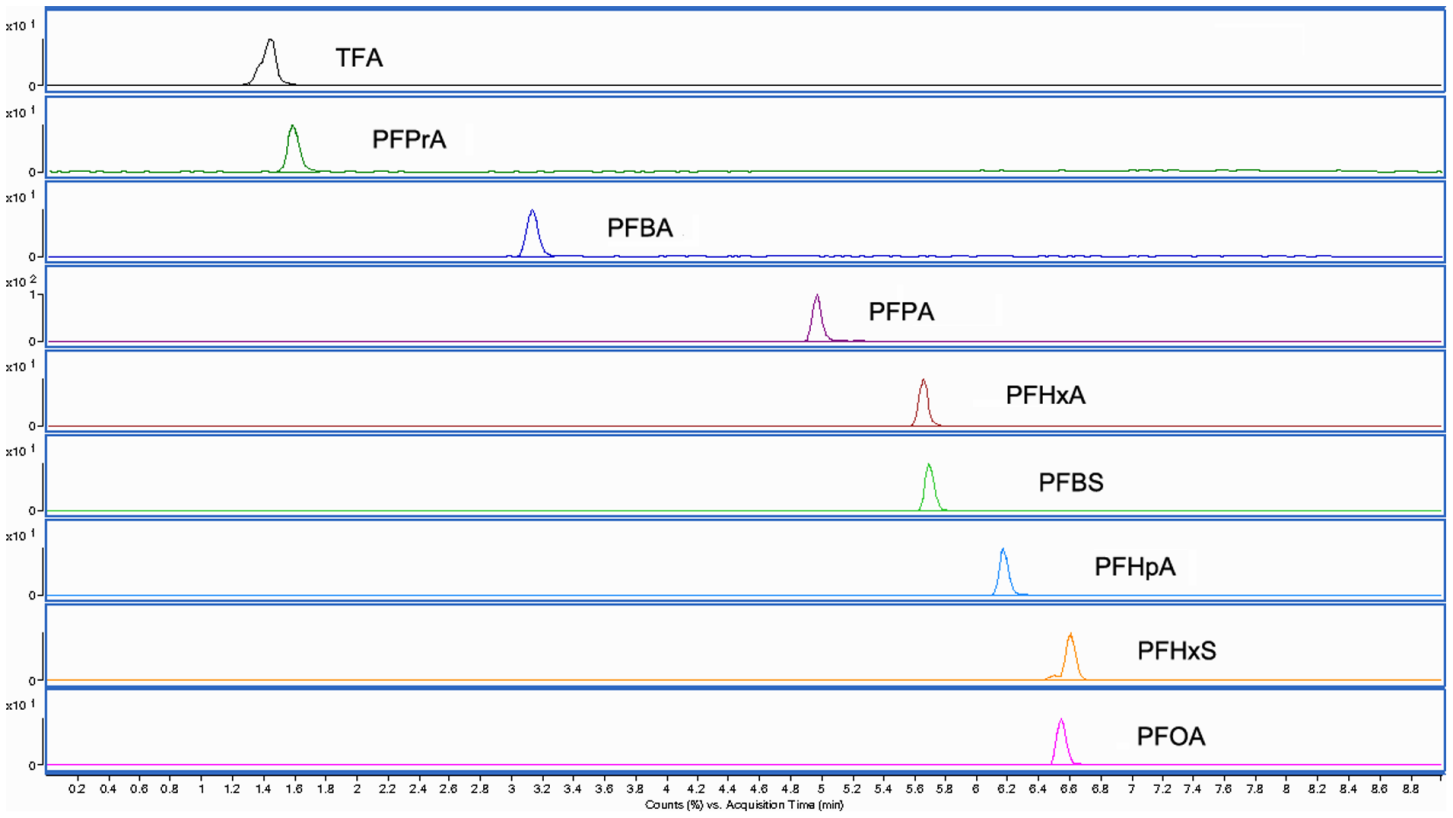
MRM spectra of standard solutions of nine kinds of PFCs.

The sample solution and standard solution were injected into the liquid chromatography tandem mass spectrometry, respectively. MRM spectra of standard solutions of nine kinds of PFCs were showed in Fig. 2. The linear regression equations between the concentration of each PFCs and peak area of characteristic peaks were built with the correlation coefficient R^2^ more than 0.99. The linear ranges of each PFCs were 0.001–1 mg/L.

Ion Chromatography. The concentrations of F^−^ and SO_4_^2−^ were determined by an anion-chromatography system (Dionex, ICS-1000, USA) consisting of a degasser, a sampler (1 mL injection volume), a guard column (AS4A-SC 4×50 mm, Dionex), a separation column (AS4A-SC 4×250 mm, Dionex), a column heater (30°C), and a conductivity detector with a suppressor. A mixture solution containing 3.5 mM Na_2_CO_3_ and 1.0 mM NaHCO_3_ as the mobile phase was delivered at a flow rate of 1.0 mL·min^−1^.The lowest detection limit of F^−^ and SO_4_^2−^ was 0.02 mg/L and 0.1 mg/L, respectively.

## Results and Discussion

### Defluorinationof PFOS by K_2_S_2_O_8_ oxidation

**Figure 3:**
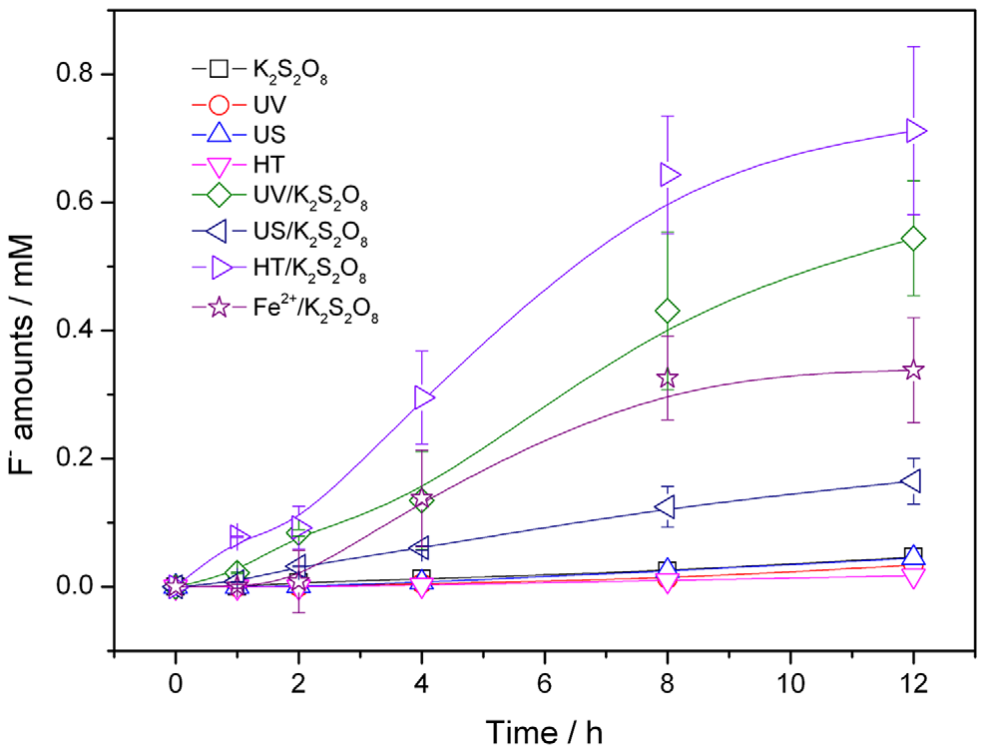
Time course of PFOS defluorination in activated K_2_S_2_O_8_ oxidation systems.

The defluorination of PFOS (100 mg/L, 0.186 mM) in each system with the initial K_2_S_2_O_8_ amount of 5 g/L (18.5 mM) were shown in Fig. 3.

The S_2_O_8_^2−^ had weak effect on the defluorination of PFOS. The defluorination efficiency (moles of F^−^ formed)/(moles of fluorine content in initial PFOS) of PFOS were only 1.45% in solo K_2_S_2_O_8_ system after 12 h, indicating that S_2_O_8_^2−^ was not an efficient oxidant for degrading PFOS at the room temperature of 20°C. In contrast, the K_2_S_2_O_8_ showed different defluorination efficiencies of PFOS in the presence of catalyst, such as UV, US, HT and Fe^2+^. It should be mentioned that these treatments had poor effects on PFOS defluorination when they are used alone. Thus, it can be concluded that the sulfate radical oxidation was mainly responsible for the defluorination of PFOS in activated K_2_S_2_O_8_ oxidation. On the basis of data (Fig. 3) of this research, the varying tendencies of F^−^ concentration were fitted using first-order kinetics equation. According to the fitting results, the apparent rate constants of F^−^ in UV/K_2_S_2_O_8_, US/K_2_S_2_O_8_, HT/K_2_S_2_O_8_ and Fe^2+^/K_2_S_2_O_8_ were 0.016, 0.004, 0.023 and 0.010 h^−1^, respectively. Thus, PFOS was defluorinated faster in HT/K_2_S_2_O_8_ system than others. The highest PFOS defluorination efficiency reached 22.52% in HT/K_2_S_2_O_8_ system after 12 h. Temperature has an important influence on free radical and its inducing oxidization reactions. The PFOS move more quickly at high temperature, which increases their probability to react with SO_4_^•−^. Thus, high temperature could not only activate S_2_O_8_^2−^ but also promote the reaction process, and HT/K_2_S_2_O_8_ system had showed better defluorination effect than other activated K_2_S_2_O_8_ oxidation systems.

**Figure 4:**
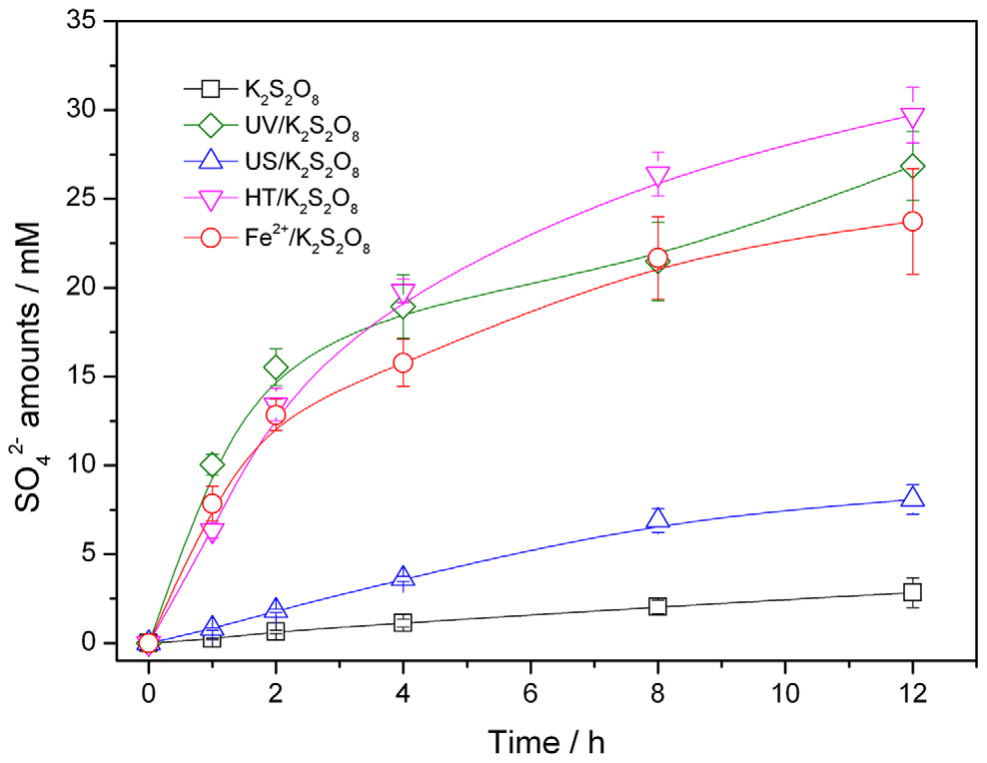
Time course of SO_4_^2−^ amounts in activated K_2_S_2_O_8_ oxidation systems.

The formation of SO_4_^2−^ in each system with the initial K_2_S_2_O_8_ amount of 5 g/L (18.5 mM) was also shown in Fig. 4.

In activated K_2_S_2_O_8_ oxidation, SO_4_^•−^ radical anions were formed from S_2_O_8_^2−^, which was then react with PFOS. The SO_4_^2−^ could be formed through two main ways. One way is that one-electron transfer from reducible agent such as PFOS, S_2_O_8_^2−^ and H_2_O to SO_4_^•−^ (total concentration = 37 mM) and the other way is sulfonic acid group deprivation to form PFOS (total concentration = 0.186 mM). By contrast, the SO_4_^2−^ generation form sulfonic acid group deprivation could be negligible, therefore we could assume that all the SO_4_^2−^ was product of S_2_O_8_^2−^.

Low formation of SO_4_^2−^ under sole K_2_S_2_O_8_ oxidization treatment, implied that S_2_O_8_^2−^ was the major oxidants during the reaction. The activated K_2_S_2_O_8_ oxidation treatment showed different production of SO_4_^2−^. Based on these data (Fig. 2), the concentrations of SO_4_^2−^ were fitted using first-order kinetics equation. According to the fitting results, the apparent rate constants of SO_4_^2−^ in UV/K_2_S_2_O_8_, US/K_2_S_2_O_8_, HT/K_2_S_2_O_8_ and Fe^2+^/K_2_S_2_O_8_ were 0.162, 0.026, 0.233 and 0.131 h^−1^, respectively. Li et al. found that K_2_S_2_O_8_ could be activated by ultrasonic irradiation and degradation rates of TCA was increased from 0.0014 to 0.396 min^−1^ with an increase in the ultrasound frequency from 50 to 400 kHz [31]. However, in present study, low frequency of US (40 KHz) could not effectively decomposes S_2_O_8_^2^− to sulfate radicals. In contrast, S_2_O_8_^2−^ was fastly converted to SO_4_^2^− under Fe2+/K_2_S_2_O_8_ treatment. However, the PFOS defluorination efficiency was only 23.50% after 12 h. One reason for this phenomenon could be that there is lower production of SO_4_^•−^ under Fe^2+^/K_2_S_2_O_8_ treatment than UV/K_2_S_2_O_8_ and HT/K_2_S_2_O_8_ treatment. Homolysis of 1 mol S_2_O_8_^2−^ produce two moles SO_4_^•−^ using heat or light but only one mole SO_4_^•−^ production using Fe^2+^ catalyst (Eq.7):
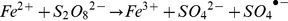
(7)


### Effect of initial solution pH

Effects of initial pH (3∼11) on defluorination of PFOS 0.186 mM (100 mg/L) under each activated K_2_S_2_O_8_ oxidation treatment was shown in Table 2.

**Table 2:** The calculated pseudo-first-order constants and defluorination efficiency (%) of PFOS with 18.5 mM persulfate in activated K_2_S_2_O_8_ oxidation systems at different initial pH values.

system	Initial pH	Rate constant (per hour)	Reaction time (h)			
			1	4	8	12
UV/K_2_S_2_O_8_	3.13	0.018	0.94	5.57	15.91	19.32
	6.91	0.016	0.70	4.27	13.63	17.21
	11.11	0.009	-	3.23	6.89	10.56
US/K_2_S_2_O_8_	3.11	0.005	-	2.47	4.90	5.82
	7.04	0.004	0.25	1.93	3.95	5.22
	10.92	0.003	-	0.98	3.10	3.51
HT/K_2_S_2_O_8_	3.11	0.025	2.18	10.56	19.96	25.71
	6.94	0.023	2.47	9.35	20.33	22.52
	10.95	0.013	0.16	4.87	10.15	15.24
Fe^2+^/K_2_S_2_O_8_	3.11	0.013	0.44	4.93	12.57	13.88
	7.12	0.010	-	4.36	10.31	10.68
	11.24	0.005	-	1.77	4.02	6.07

**Figure 5:**
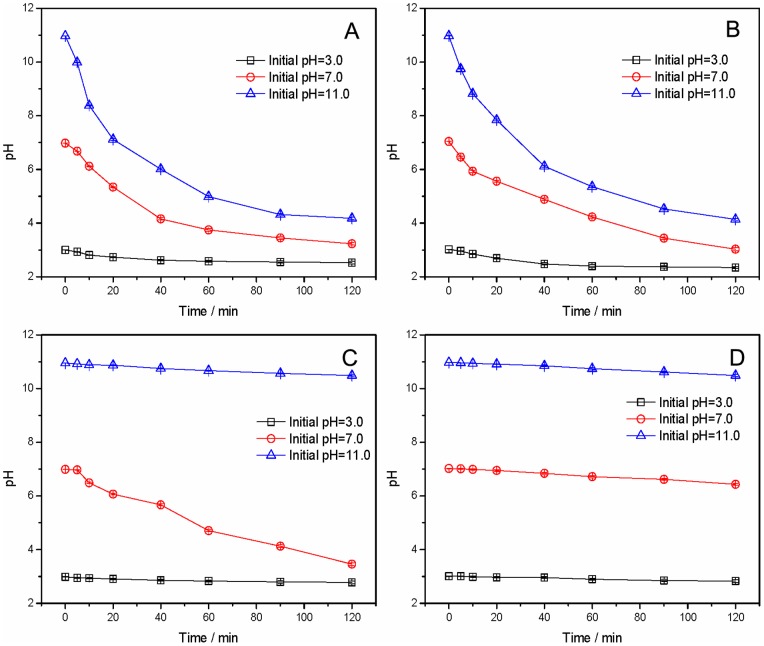
Change of pH of K_2_S_2_O_8_ solution during reaction process in activated oxidation systems. (A) UV/K_2_S_2_O_8_; (B) HT/K_2_S_2_O_8_; (C) Fe^2+^/K_2_S_2_O_8_; (D) US/K_2_S_2_O_8_.

During the experimental runs, pH of the PFOS solution dropped as the reaction progressed due to formation of lager amount of SO_4_^2−^. Change of pH in K_2_S_2_O_8_ solution during reaction process in activated oxidation systems was shown in Fig. 5. With the reaction going on, pH of the solution dropped, and showed different changing tendency with different initial pH in each activated persulfate oxidation system. The pH of US/K_2_S_2_O_8_ system slowly decreased as compared with other three systems. It inferred that US had a weaker activating capability than UV, HT and Fe^2+^. Moreover, there was no obvious change in pH in Fe^2+^/K_2_S_2_O_8_ system in 120 min at initial pH of 11.0. Thus, a fixed initial pH was just maintained at the initial stage of PFOS defluorination in UV/K_2_S_2_O_8_ and HT/K_2_S_2_O_8_ systems, and later reaction was conducted under the acidic condition. Even so, PFOS defluorination was varied with initial pH under each activated K_2_S_2_O_8_ oxidation treatment. As shown in Table 2, the PFOS defluorination efficiency decreased with the increase of initial pH under all treatment, especially for Fe^2+^/K_2_S_2_O_8_.

The PFOS defluorination efficiency increased with deceasing of solution pH, because additional sulfate radicals were formed due to acid catalyzation [32]. In addition, sulfate radicals may react with OH^−^ to form more hydroxyl radical under alkaline conditions [28], as shown by Eq. (8) below:
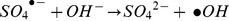
(8)


The generated hydroxyl radicals generally attack organic molecules through the H-atom abstraction to form water. However, PFOS contain no hydrogen to be abstracted, therefore hydroxyl radicals have a very poor reactivity with PFOS in aqueous solution and slow down the PFOS decomposition rate. Thus, alkaline conditions were unfavorable for the defluorination of PFOS by sulfate radicals. The pH of PFOS solution decrease during the defluorination process, but high initial concentration of OH^−^ need to consume SO_4_^•−^, which could weaken the oxidant effect of activated K_2_S_2_O_8_ oxidization. Thus, the PFOS defluorination efficiency decreased with the increase of initial pH under all treatment. In addition, compared with UV/K_2_S_2_O_8_ and HT/K_2_S_2_O_8_ system, the more decrease in PFOS defluorination under Fe^2+^/K_2_S_2_O_8_ treatment under alkaline conditions could be explained by the formation of Fe(OH)_2_ which cause the deactivation of catalyst.

### Effect of S_2_O_8_^2−^ amounts

**Figure 6:**
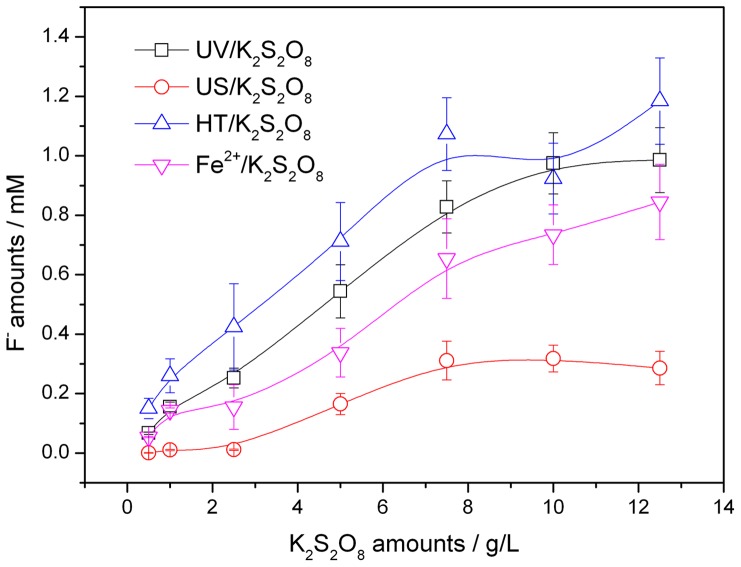
Effect of K_2_S_2_O_8_ amounts on defluorination of PFOS in activated K_2_S_2_O_8_ oxidation systems.

The effect of initial S_2_O_8_^2−^ amount (0–12.5 g/L) on defluorination of PFOS (100 mg/L) under each activated K_2_S_2_O_8_ oxidation treatment was shown in Fig. 6.

The defluorination efficiency increased under all treatments when the initial amount of S_2_O_8_^2−^ was increased. However, further increase in the initial amount of S_2_O_8_^2−^ resulted in saturation, there is no further increase in the PFOS defluorination efficiency. The saturated concentrations of S_2_O_8_^2−^ were different with the treatment used. Hori et al. reported that sulfate radicals react with S_2_O_8_^2−^ or themselves (Eq.9, 10), In addition, excess SO_4_^•−^ can also react with H_2_O (Eq.11) [27]:
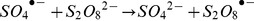
(9)

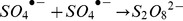
(10)

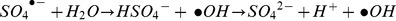
(11)


The SO_4_^•−^/S_2_O_8_^2−^, SO_4_^•−^/SO_4_^•−^ and SO_4_^•−^/H_2_O reaction could compete with the SO_4_^•−^/PFOS for electrons [28]. Thus, a large amounts of SO_4_^•−^ were produced along with high concentration of K_2_S_2_O_8_ under activated K_2_S_2_O_8_ oxidation treatment. The scavenging reactions by SO_4_^•−^ themselves and with the remaining S_2_O_8_^2−^ may become significant to decrease PFOS defluorination efficiency. In this case, the systems might have generated much S_2_O_8_^•−^ and •OH when sulfate radicals too excessive to react with PFOS. Oxidation by S_2_O_8_^•−^ and •OH are not effective for PFOS and other PFCs because of low rates of reaction at reasonable S_2_O_8_^•−^ and •OH and their lower oxidation capacity compared with SO_4_^•−^. Therefore, further increase in S_2_O_8_^2−^ concentration resulted in production of larger amounts of SO_4_^•−^. The scavenging reactions by SO_4_^•−^ themselves and with the excess S_2_O_8_^2−^ or H_2_O might become significant to decrease in PFOS defluorination efficiency.

### PFOS defluorination by-products

**Figure 7:**
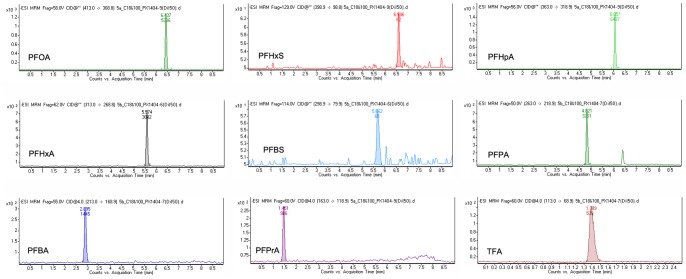
MRM spectra of assigned ionic compounds in the degraded solutions after 4h by UV/K_2_S_2_O_8_ oxidation.

We measured the concentrations of PFOS and its intermediates under UV/K_2_S_2_O_8_ treatment during UV irradiation. Fig. 7 shows the MRM spectra of assigned ionic compounds in the degraded solutions after 4 h by UV/K_2_S_2_O_8_ oxidation.

**Figure 8:**
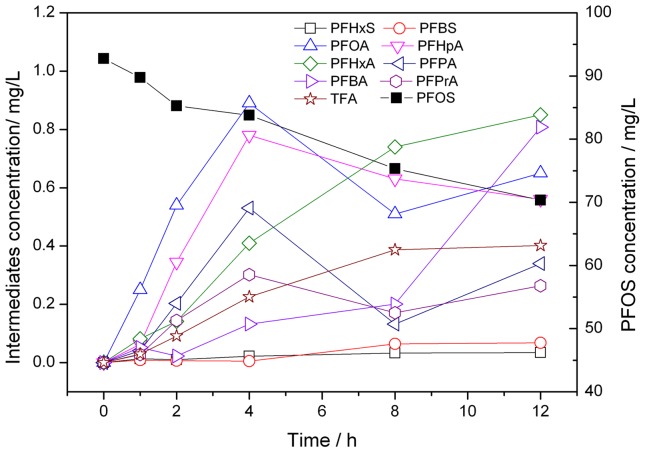
Changes in the concentration of PFOS and intermediates through the PFOS defluorination in UV/K_2_S_2_O_8_ system.

The concentrations of PFOS and the intermediates at different irradiation time were determined using external standard method. The results were shown in Fig. 8.

The concentrations of PFOA, PFHpA and PFPA first increased and then decreased during the reaction process, and the concentrations of PFHxA, PFBA, PFPrA and TFA gradually increased with the increasing of time. As intermediates, PFCAs were not only the hydrolysis product but also the reactant of following reactions. The formed perfluorinated carboxylic acid undergo a further degradation and shortening of the perfluorocarbon chain. Based on the reaction dynamics of continuous reaction, the concentration of PFCAs should first increase and then decreased during the reaction process. From the results, it can infer that PFHxA, PFBA, PFPrA and TFA could not reached the maximum at 12 h. On the other hand, there is low concentration of PFHxS and PFBS were maintained at the constant concentration over time. The formation of trace levels of PFHxS and PFBS would be due to the recombination of CF_3_(CF_2_)_5_·and CF_3_(CF_2_)_3_·with SO_3_^−^ group [8].

Because of the lack of standards of some compounds we could not measure their actual concentration. The unknown fluorines are those not to be confirmed by above analysis. Thus, the irradiation-time dependence of the mass balance of fluorine was also studied in this study. The mass concentration of the inorganic fluorine in the solutions was analyzed using IC at different reaction time. The organic fluorine referred mainly to the fluorine in PFOS and intermediates which had been detected by LC/MS/MS and their mass concentration was calculated from mass ratio between fluorine and parent PFCs (Eq.12).
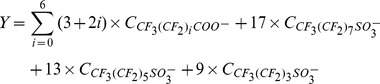
(12)


The result of analysis of mass balance of fluorine was given in Table 3. The sum of the fluorine in PFOS, PFCAs and inorganic fluorine accounted for greater than 95% of the fluorine from the degraded PFOS at any point in time during UV/K_2_S_2_O_8_ system. According to the results of analyses of the intermediates and the mass balance of fluorine, it can be inferred that PFCAs were the main intermediates.

**Table 3:** Irradiation-time dependence of fluorine element mass balance during decomposition of PFOS in UV/K_2_S_2_O_8_ systems.

Time (h)	Organic fluorine (mg/L)		Inorganic fluorine (mg/L)	Unknown fluorine (mg/L)
	PFOS	Intermediates	F^−^	
0	60	0	0	0
1	58.34	0.37	0.42	0.87
2	55.42	0.99	1.6	1.99
4	54.46	2.15	2.57	0.82
8	48.96	1.84	8.19	1.01
12	45.72	2.53	10.34	1.41

### PFOS defluorination mechanisms

Previous investigations have provided that PFOS could lose electrons to anode or strong oxidizers, but the mechanism was still in controversy. Kimberly et al. found that an alternative oxidation mechanism is the direct transfer of electrons from PFOS to the BDD (Boron-Doped Diamond) anode as shown in Eq.13 [19].
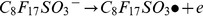
(13)


Lin et al. reported that hydroxyl radicals attack the perfluoro-anion and become quenched into hydroxide ions, leaving perfluorinated radicals for continuing chain reactions that lead to further decomposition [20]. Liu et al. found that the first oxidative attack of PFOS by permanganate may have occurred at the C-S and C-C bonds, which released the -CF_2_ units and oxidation product of SO_4_^2−^ to form the shorter chain perfluoroalkyl sulfonates and perfluorochemical products subject to further transformation reactions such as hydrolysis [2].

As another most extensive and typical of PFCs, PFOA oxidation mechanism with SO_4_^•−^ have previously reported. Both Hori et al. and Lee et al. reported that the PFOA oxidation mechanism with SO_4_^•−^ was described as follows (Eq.14–18) [27], [28]:
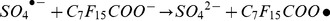
(14)

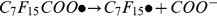
(15)

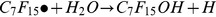
(16)

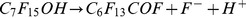
(17)

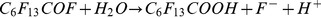
(18)


**Figure 9:**
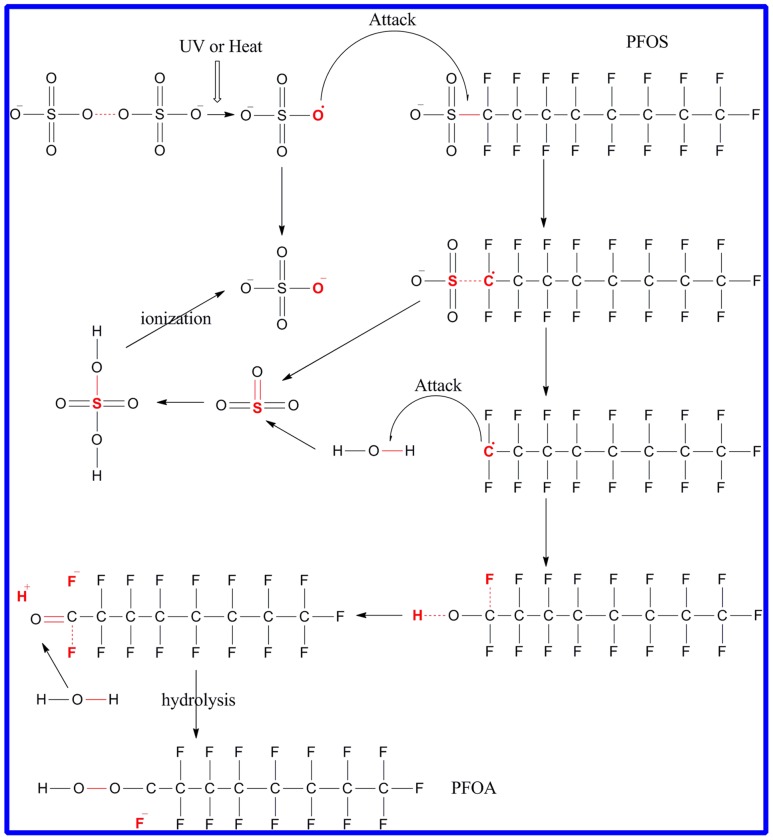
Possible defluorination pathways of PFOS in the activated K_2_S_2_O_8_ oxidation systems.

Based on the results discussed above, the mechanism of oxidative defluorination of PFOS with sulfate radicals could be illuminated. The defluorination of PFOS was as Fig. 9: The oxidation potential of SO_4_^•−^ is 2.5–3.1eV, while the C-F bond in PFOS is the most oxidation resistant bond (E0 = 3.6 eV) and is difficult to be dissociated by SO_4_^•−^. Therefore, the first oxidative attack of PFOS by SO_4_^•−^ may have occurred at the C-S bonds, therefore, SO_4_^•–^ oxidize PFOS to form C_8_F_17_•, the unstable C_8_F_17_• may react with H_2_O to form unstable C_8_F_17_OH, which undergo HF elimination to form C_7_F_15_COF. Moreover, C_7_F_15_COF further undergo hydrolysis, resulting in the formation of short chain PFOA(C_7_F_15_COOH). PFOA is first formed by dissociation of two fluorine of PFOS, and long-chain PFCAs are decomposed stepwise to form short-chain perfluorocarboxylic acid such as PFHpA, PFHxA, PFPA and PFBA are formed successively by similar reaction. Moreover, previous investigations have provided that PFOA and other short-chain perfluorocarboxylic acid could be effectively degraded by sulfate radicals [28]. Hori et al. found that the defluorination of PFOA (1.35 mM) reached 73.8% in UV/K_2_S_2_O_8_ system after 12 h of irradiation. And they also reported that the defluorination of PFOA (0.374 mM) was 77.5% in HT/K_2_S_2_O_8_ system at 80°C after 12 h [33]. In addition, Lee et al. found that short-chain PFCAs are easier to degrade and mineralize than long-chain PFCAs [28]. Thus, the SO_4_^•−^/PFCAs reaction could compete with the SO_4_^•−^/PFOS reaction. Our results show that PFOS was much more stable and more difficult to be defluorinated by sulfate radicals. It can be inferred that SO_4_^•−^/PFCAs could be more likely to occurred than the SO_4_^•−^/PFOS.

## Conclusion

The S_2_O_8_^2−^ showed weak effect on defluorination of PFOS while sulfate radicals could oxidatively decompose PFOS. The defluorination efficiencies were varied with the K_2_S_2_O_8_ oxidation activated by different treatment. The defluorination performance of treatments follow the orders: HT/K_2_S_2_O_8_ > UV/K_2_S_2_O_8_ > Fe^2+^/K_2_S_2_O_8_ > US/K_2_S_2_O_8_. Acidic conditions was favorable for the defluorination of PFOS by sulfate radicals. The defluorination efficiency increased with increases of the initial amount of S_2_O_8_^2−^ under all activated K_2_S_2_O_8_ oxidation treatment. However, further increase in the initial amounts of S_2_O_8_^2−^ did not resulted in further increase in PFOS defluorination efficiency. The main intermediates during the degradation of PFOS were short-chain PFCs. Oxidization and hydrolysiswere the main defluorination mechanism of PFOS. PFOS was first lost electron to sulfate radicals and then desulfonated to C_8_F_17_ radical. During the hydrolytic reaction, long-chain PFCAs are decomposed stepwise to form short-chain PFCAs.

## Supporting Information

Figure S1 The effects of low temperature (0°C) on the defluorination of PFOS (0.186 mM) with S_2_O_8_^2−^ (18.5 mM) and Fe^2+^ (3 mM) in ice water were investigated. IC spectra of F^−^, S_2_O_8_^2−^ and SO_4_^2−^ before and after reaction were shown in Fig. S1. The results showed that no F^−^ and SO_4_^2−^ was observed after 20 min, and there was less change in concentration of S_2_O_8_^2−^. Thus, it could be considered that S_2_O_8_^2−^ was stable in iced water, and the ice water could quench the formation SO_4_^•−^. Due to the time for sampling was controlled in 10 min, we can considered that this method for quenching reaction could ensure the accuracy of the F^−^ detection.
(TIF)

## References

[1] Kannan K, Corsolini S, Falandysz J, Fillmann G, Kumar KS, et al.. (2004) Perfluorooctane sulfonate and related fluorochemicals in human blood from several countries. Environmental Science & Technology 38, 4489–4495. 10.1021/es0493446 15461154

[2] Liu CS, Shih K, Wang F (2012) Oxidative decomposition of perfluorooctane sulfonate in water by permanganate. Separation and Purification Technology 87, 95–100.

[3] Sinclair E, Kannan K (2006) Mass loading and fate of perfluoroalkyl surfactants in wastewater treatment plants. Environmental Science & Technology 40: 1408–1414. 16568749 10.1021/es051798v

[4] Schultz MM, Higgins CP, Huset CA, Luthy RG, Barofsky DF, et al (2006) Fluorochemical mass flows in a municipal wastewater treatment facility. Environmental Science & Technology 40: 7350–7357. 17180988 10.1021/es061025m PMC2556954

[5] Hollingsworth J, Sierra-Alvarez R, Zhou M, Ogden KL, Field JA (2005) Anaerobic biodegradability and methanogenic toxicity of key constituents in copper chemical mechanical planarization effluents of the semiconductor industry. Chemosphere 59: 1219–1228. 15857633 10.1016/j.chemosphere.2004.11.067

[6] Key BD, Howell RD, Criddle CS (1998) Defluorination of organofluorine sulfur compounds by Pseudomonas sp. strain D2. Environmental Science & Technology 32: 2283–2287.

[7] Horst FS, Roland JW (2005) Stability of fluorinated surfactants in advanced oxidation processes – A follow up of degradation products using flow injection-mass spectrometry, liquid chromatography-mass spectrometry and liquid chromatography-multiple stage mass spectrometry. Journal of Chromatography A 1082: 110–119. 16038200 10.1016/j.chroma.2005.02.070

[8] Yamamoto T, Noma Y, Sakai S, Shibata Y (2007) Photo degradation of perfluorooctanesulfonate by UV irradiation in Water and Alkaline 2-Propanol. Environmental Science & Technology 41: 5660–5665. 17874770 10.1021/es0706504

[9] Herrera V, Alvarez R, Somogyi A, Jacobsen N, Wysocki V, et al (2008) Reductive defluorination of perfluorooctanesulfonate. Environmental Science & Technology 42: 3260–3264. 18522103 10.1021/es702842q

[10] Park H (2010) Photolysis of aqueous perflurooctanoate and perfluorooctane sulfonate. Revue Roumaine de Chimie 55: 611–619.

[11] Park H, Vecitis CD, Cheng J, Mader BT, Hoffmann MR (2009) Reductive defluorination of aqueous perfluorinated alkyl surfactants: Effects of ionic headgroupand chain length. Journal of Physical Chemistry A 113: 690–696. 10.1021/jp807116q 19123849

[12] Moriwaki H, Takagi Y, Tanaka M, Tsuruho K, Okitsu K, et al (2005) Sonochemical decomposition of perfluorooctane sulfonate and perfluorooctanoic acid. Environmental Science & Technology 39: 3388–3392. 15926594 10.1021/es040342v

[13] Vecitis CD, Park H, Cheng J, Mader BT, Hoffmann MR (2008) Enhancement of perfluorooctanoate and perfluorooctanesulfonate activity at acoustic cavitation bubble interfaces. Journal of Physical Chemistry C 112: 16850–16857. 10.1021/jp801081y 18447373

[14] Cheng J, Vecitis CD, Park H, Mader BT, Hoffmann MR (2008) Sonochemical degradation of perfluorooctane sulfonate (PFOS) and perfluorooctanoate (PFOA) in landfill groundwater: Environmental matrix effects. Environmental Science & Technology 42: 8057–8063. 19031902 10.1021/es8013858

[15] Vecitis CD, Park H, Cheng J, Mader BT, Hoffmann MR (2008) Kinetics and mechanism of the sonolytic conversion of the aqueous perfluorinated surfactants, perfluorooctanoate (PFOA), and perfluorooctane sulfonate (PFOS) into inorganic products. Journal of Physical Chemistry A 112: 4261–4270. 10.1021/jp801081y 18447373

[16] Cheng J, Vecitis C D, Park H, Mader BT, Hoffmann MR (2010) Sonochemical degradation of perfluorooctane Sulfonate (PFOS) and perfluorooctanoate (PFOA) in groundwater: Kinetic Effects of Matrix Inorganics. Environmental Science & Technology 44: 445–450. 19950930 10.1021/es902651g

[17] Vecitis CD, Wang Y, Cheng J, Park H, Mader BT, et al (2010) Sonochemical degradation of perfluorooctanesulfonate in aqueous film-forming foams. Environmental Science & Technology 44: 432–438. 19961151 10.1021/es902444r

[18] Hori H, Nagaoka Y, Yamamoto A, Sano T, Yamashita N, et al (2006) Efficient decomposition of environmentally persistent perfluorooctanesulfonate and related fluorochemicals using zerovalent iron in subcritical water. Environmental Science & Technology 40: 1049–1054. 16509356 10.1021/es0517419

[19] Carter KE, Farrell J (2008) Oxidative destruction of perfluorooctane sulfonate Using Boron-Doped Diamond Film Electrodes. Environmental Science & Technology 42: 6111–6115. 18767674 10.1021/es703273s

[20] Lin AY, Panchangama SC, Chang C, Andy Hong PK, Hsueh H (2012) Removal of perfluorooctanoic acid and perfluorooctane sulfonate via ozonation under alkaline condition. Journal of Hazardous Materials 243: 272–277. 23131499 10.1016/j.jhazmat.2012.10.029

[21] Lee YC, Lo SL, Chiueh PT, Liou YH, Chen ML (2010) Microwave-hydrothermal decomposition of perfluorooctanoic acid in water by iron-activated persulfate oxidation. Water research 44: 886–892. 19879622 10.1016/j.watres.2009.09.055

[22] Huang KC, Couttenye RA, Hoag GE (2007) Kinetics of heat assisted persulfate oxidation of methyl tert-butyl ether (MTBE). Chemosphere 49: 413–420. 10.1016/s0045-6535(02)00330-2 12365838

[23] Liang C, Wang ZS, Bruell CJ (2007) Influence of pH on persulfate oxidation of TCE at ambient temperatures. Chemosphere 66: 106–113. 16814844 10.1016/j.chemosphere.2006.05.026

[24] Anipsitakis GP, Dionysiou DD, Gonzalez MA (2006) Cobaltmediated activation of peroxymonosulfate and sulfate radical attack on phenolic compounds. Implications of chloride ions. Environmental Science & Technology 40: 1000–1007. 16509349 10.1021/es050634b

[25] Liang CJ, Bruell CJ, Marley MC, Sperry KL (2003) Thermally activated persulfate oxidation of trichloroethylene (TCE) and 1,1,1-trichloroethane (TCA) in aqueous systems and soil slurries. Soil&Sediment Contamination 12: 207–228.

[26] Waldemer RH, Tratnyek PG, Johnson RL, Nurmi JT (2007) Oxidation of chlorinated ethenes by heat-activated persulfate: Kinetics and products. Environmental Science & Technology 41: 1010–1015. 17328217 10.1021/es062237m

[27] Hori H, Yamamoto A, Hayakawa E, Taniyasu S, Yamashita N, et al (2005) Efficient decomposition of environmentally persistent perfluorocarboxylic acids by use of persulfate as a photochemical oxidant. Environmental Science & Technology 39: 2383–2388. 15871280 10.1021/es0484754

[28] Lee YC, Lo SL, Chiueh PT, Chang DG (2009) Efficient decomposition of perfluorocarboxylic acids in aqueous solution using microwave-induced persulfate. Water Research 43: 2811–2816. 19443010 10.1016/j.watres.2009.03.052

[29] Lee YC, Lo SL, Chiueh PT, Liou YH, Chen ML (2010) Microwave-hydrothermal decomposition of perfluorooctanoic acid in water by iron-activated persulfate oxidation. Water research 44: 886–892. 19879622 10.1016/j.watres.2009.09.055

[30] Lee YC, Lo SL, Kuo J, Lin YL (2012) Persulfate oxidation of perfluorooctanoic acid under the temperatures of 20–40°C. Chemical Engineering Journal 198: 27–32.

[31] Li BZ, Li L, Lin K, Zhang W, Lu S, et al (2013) Removal of 1,1,1-trichloroethane from aqueous solution by a sono-activated persulfate process. Ultrasonics Sonochemistry 20: 855–863. 23266439 10.1016/j.ultsonch.2012.11.014

[32] Liang C, Wang ZS, Bruell CJ (2007) Influence of pH on persulfate oxidation of TCE at ambient temperatures. Chemosphere 66: 106–113. 16814844 10.1016/j.chemosphere.2006.05.026

[33] Hori H, Nagaoka Y, Murayama M, Kutsuna S (2008) Efficient decomposition of perfluorocarboxylic acids and alternative fluorochemical surfactants in hot water. Environmental Science & Technology 42: 7438–7443. 18939583 10.1021/es800832p

