# Supplementary figures and images for: Defluorination of Aqueous Perfluorooctanesulfonate by Activated Persulfate Oxidation
Source: PLoS One. 2013 Oct 7;8(10):e74877. doi: 10.1371/journal.pone.0074877 (PMC3792066; doi:10.1371/journal.pone.0074877)

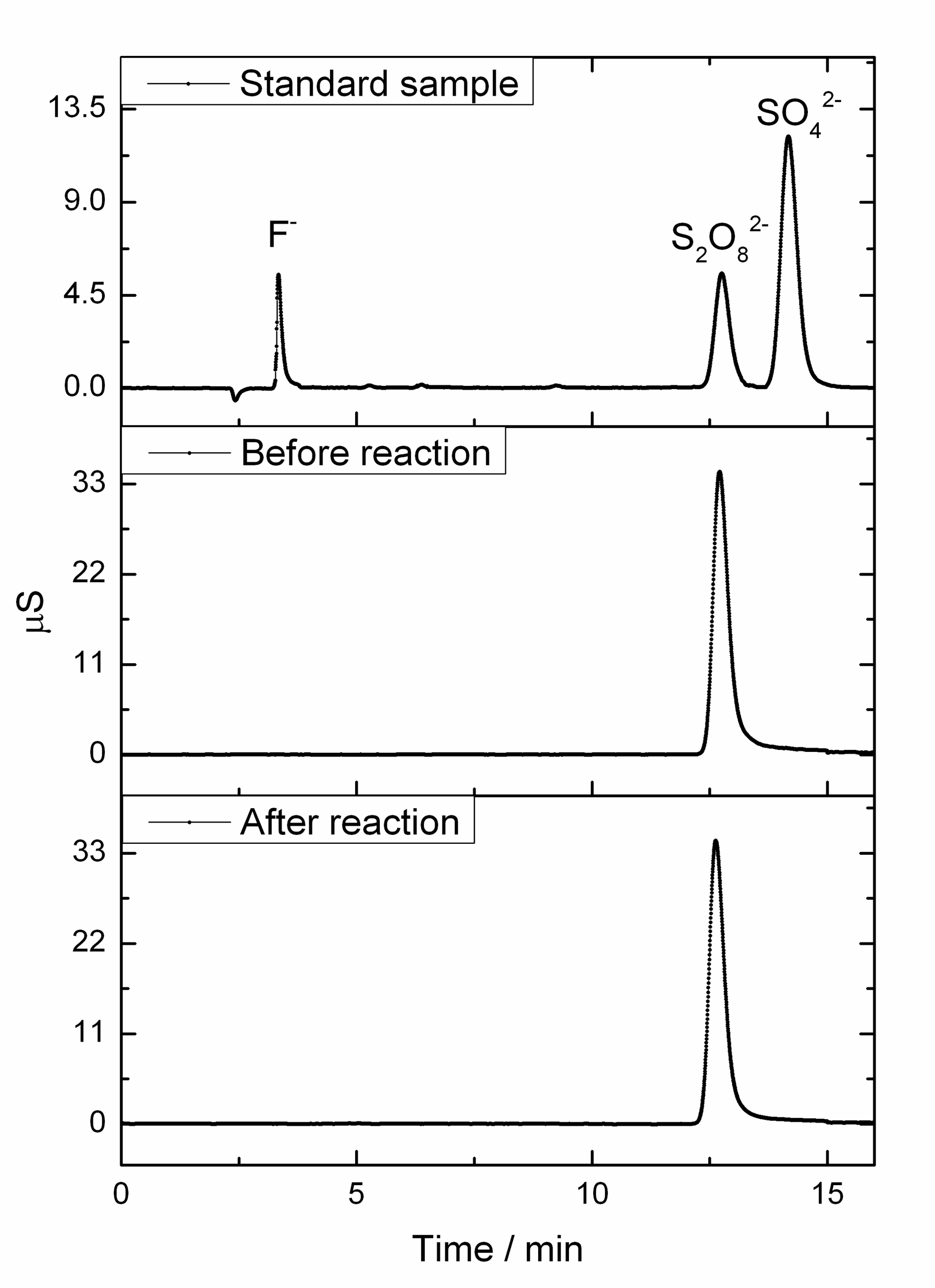

Supplement: Figure S1 — The effects of low temperature (0°C) on the defluorination of PFOS (0.186 mM) with S2O8 2− (18.5 mM) and Fe2+ (3 mM) in ice water were investigated. IC spectra of F−, S2O8 2− and SO4 2− before and after reaction were shown in Fig. S1. The results showed that no F− and SO4 2− was observed after 20 min, and there was less change in concentration of S2O8 2−. Thus, it could be considered that S2O8 2− was stable in iced water, and the ice water could quench the formation SO4 •−. Due to the time for sampling was controlled in 10 min, we can considered that this method for quenching reaction could ensure the accuracy of the F− detection. (TIF) [file pone.0074877.s001.tif]
